# M1/M2 Macrophage Polarity in Normal and Complicated Pregnancy

**DOI:** 10.3389/fimmu.2014.00606

**Published:** 2014-11-24

**Authors:** Mary B. Brown, Maria von Chamier, Ayman B. Allam, Leticia Reyes

**Affiliations:** ^1^D. H. Barron Reproductive and Perinatal Biology Research Program, Department of Infectious Disease and Pathology, College of Veterinary Medicine, University of Florida, Gainesville, FL, USA

**Keywords:** macrophages, M1/M2, normal pregnancy, complicated pregnancy, uterine atherosis, spiral artery, chorioamnionitis, spontaneous abortion

## Abstract

Tissue macrophages play an important role in all stages of pregnancy, including uterine stromal remodeling (decidualization) before embryo implantation, parturition, and post-partum uterine involution. The activation state and function of utero-placental macrophages are largely dependent on the local tissue microenvironment. Thus, macrophages are involved in a variety of activities such as regulation of immune cell activities, placental cell invasion, angiogenesis, and tissue remodeling. Disruption of the uterine microenvironment, particularly during the early stages of pregnancy (decidualization, implantation, and placentation) can have profound effects on macrophage activity and subsequently impact pregnancy outcome. In this review, we will provide an overview of the temporal and spatial regulation of utero-placental macrophage activation during normal pregnancy in human beings and rodents with a focus on more recent findings. We will also discuss the role of M1/M2 dysregulation within the intrauterine environment during adverse pregnancy outcomes.

## Introduction

Macrophages within the maternal–fetal compartment have been a research focus for over 30 years. Both maternal and fetal derived macrophages play an important role in all stages of pregnancy. These cells support a variety of processes essential for successful pregnancy such as remodeling of the uterine connective tissues and blood vessels, regulation of trophoblast (fetal cell) implantation, immune-tolerance toward fetal antigens, immunomodulation of neighboring leukocytes, and initiation of parturition (1–5). All of these functions are manifestations of macrophage polarity or state of activation, which at the most basic level has been referred to as M1 and M2. M1 macrophages display the capacity to present antigen, produce IL-12, IL-23, reactive oxygen species (6, 7), and skew T cell responses toward a TH1 or cell mediated immune response (8). In contrast, M2 macrophages participate in tissue remodeling, have immunosuppressive qualities, and promote TH2 or antibody mediated immune responses (6). In essence, M1/M2 activities arise from arginine metabolism via two enzymatic pathways (iNOS and arginase) that down regulate each other (7, 9). The factors that influence which pathway dominates are based on the surrounding signals that the macrophage receives as well as the available arginine pool (7, 10). Thus, macrophage function, which is a manifestation of the cell’s polarization or activation state, is ultimately decided by the surrounding milieu.

The maternal–fetal interface is a unique environment in that it comprises three distinct compartments: the placenta (fetal origin), and maternal origin (endometrium or decidua, and myometrium), which are infiltrated with fetal cells. Furthermore, these compartments undergo dramatic changes in architecture and leukocyte composition as gestation progresses. These changes are necessary for placental development and tolerance of foreign fetal antigens. Macrophages are present in all compartments and during all stages of pregnancy (implantation, placentation, fetal growth, and parturition) (11). Successful pregnancy requires that the macrophage activation state remain regulated throughout pregnancy. Indeed, inappropriate macrophage polarization within the maternal–fetal compartments is associated with spontaneous abortion or miscarriage (12), inadequate remodeling of the uterine vessels during placentation (12–14), and intrauterine parasitic infections (15). In this review, we provide an overview of M1/M2 dynamics relevant to the maternal–fetal interface, the temporal and spatial changes in macrophage M1/M2 within the maternal and fetal compartments during normal pregnancy, and imbalanced M1/M2 dynamics associated with complicated pregnancies.

## M1 and the Many Shades of M2

Macrophages display divergent phenotypes that were originally described as M1or M2 polarity (7). M1 refers to the classically activated macrophage whereby the cell displays the capacity to present antigen, produce IL-12, IL-23, and reactive oxygen species (6, 7). M1 polarized macrophages are more effective at antimicrobial killing and skewing T cell responses toward a TH1 or cell mediated immune response (8). External stimuli known to promote M1 polarization include toll-like receptor (TLR4) agonists such lipopolysaccharide (LPS) from *E. coli*, IFN-γ, TNF-α, and GM-CSF (7, 16). In contrast, M2 polarized cells (alternatively activated cells) display abundant expression of mannose and scavenger receptors and produce IL-10 and TGF-β (6). M2 macrophages participate in tissue remodeling, have immunosuppressive qualities, and promote TH2 or antibody mediated immune responses (6). Stimuli that induce M2 polarization include IL-4, IL-10, IL-13, IL-33, TGF-β, and G-CSF (7, 16–19). Because of the broad range of activities carried out by M2 macrophages, Mantovani et al. (6) later proposed that these cells be further divided into M2a (induced by IL-4 and IL-13), M2b (macrophages exposed to immune complexes or toll-like receptor agonists), and M2c (induced by IL-10 and glucocorticoid hormones). These subcategories were based on both the type of agonists responsible for triggering their differentiation and the distinct functional profile induced by these agonists. For example, M2a cells display the alternatively activated phenotype typically attributed to M2 cells. M2b confers a Type II phenotype, because it promotes TH2 responses and produces both pro- and anti-inflammatory cytokines (TNF-α, IL-1, IL-6, IL-10^high^, and IL-12^low^) (6). M2c confers an M2 macrophage that produces IL-10, TGF-β, and extracellular matrix components (6).

At the most fundamental level, M1/M2 polarity is driven by arginine metabolism and available arginine within the extracellular milieu. In essence, M1 is a product of iNOS pathway, which drives the conversion of arginine to citrulline and NO, whereas M2 is a product of the arginase pathway in which arginine is hydrolyzed into ornithine and urea (7, 20). iNOS and arginase pathways are antagonistic, not only do they compete for arginine but also arginase directly inhibits NO synthase 2 (NOS2) (10). Although M2 is defined by the arginase pathway (7, 9), in Mantovani et al.’s designation, M2b develops independent of arginase activity, while M2a and M2c still require arginase activity (6).

A distinctive feature of the iNOS pathway is that its activation requires extracellular arginine, even if an adequate level of intracellular arginine is already present (21). Without sufficient arginine iNOS is blocked at the translational level and NO is not produced even in the presence of IFN-γ or LPS. Under these conditions, the macrophage does not acquire an M1 phenotype, but still produces TNF-α in response to IFN-γ or LPS (21). This condition bears a resemblance to M2b macrophages (6). This dependency upon extracellular arginine reserves may be particularly important during pregnancy since placental tissues are rich in arginase I and II (22), and therefore, placental extracellular arginine levels may actually be too low for induction of M1 macrophages.

Several studies have attempted to characterize the functional state of macrophages at the maternal–fetal interface during different stages of a healthy pregnancy (Table 1). These include transcriptome analysis (23, 24), methylation profiling (25), characterization of cell surface markers (26–28), and immunohistochemistry (29). When viewed in the context of M1 and M2, the results from these studies suggest that M2 macrophages or M2 subgroups are the predominant phenotype in the decidua with a smaller subset of macrophages bearing some characteristics of M1 or M2b (24). One drawback is that most of these studies analyzed macrophages out of their *in situ* context so the local factors responsible for triggering the differentiation of these macrophage subsets are still unknown.

**Table 1:** **Summary of recent studies that characterized M1/M2 status of macrophages within the maternal–fetal interface during healthy pregnancy**.

First author	Year	Gestation at sampling (weeks)	Methods	Results	Reference
Laskarin	2005	6–8 Term	Immunohistochemistry, costaining for CD14 decidual and placental macrophages (Mϕ) and mannose receptor (M2 biomarker)	Confirmed that decidual and placental Mϕ express mannose receptor *in vivo*	(29)
				CD14^+^ decidual Mϕ surround endometrial glands in early pregnancy	
				50% Of CD14^+^ Mϕ in term decidua and villi (Hofbauer) express mannose receptor	
Repnik	2008	9–18 19–23 Term	Flow cytometry of decidual Mϕ	Decidual Mϕ from early/mid gestation have higher CD80, CD86, and HLA-DR expression than term Mϕ	(26)
				Mϕ in decidua basalis express CD105, DC-SIGN, and mannose receptor	
Gustafsson	2008	7–11	RNA Microarray analysis of CD14^+^ Mϕ isolated from decidua and peripheral blood	The majority of genes upregulated in decidual Mϕ were functionally related to immunomodulation and tissue remodeling consistent with an activated M2 phenotype	(23)
Houser	2011	6–12	Flow cytometry and RNA microarray of CD14^+^ Mϕ subsets isolated from decidua basalis and peripheral blood	Identified 2 decidual Mϕ subsets: CD11c^HI^ and CD11c^LO^	(24)
				CD11c^LO^ decidual Mϕ express genes associated with extracellular matrix formation, muscle regulation, and tissue growth	
				CD11c^HI^ decidual Mϕ express genes associated with lipid metabolism and inflammation	
Svensson	2011	7–12	Phenotypic characterization of decidual Mϕ by flow cytometry RNA microarray on *in vitro* differentiated Mϕ cultures	Identified 2 decidual Mϕ subsets: ICAM-3^HI^ and ICAM-3^LO^ ICAM-3 expression on decidual Mϕ positively correlated with CD11c expression as reported by Houser and colleagues M-CSF/IL-10 *in vitro* differentiated Mϕ and decidual Mϕ showed similar cytokine secretion patterns (↑IL-10, IL-6, TNF, and CCL4)	(27)
Kim	2012	Term	Methylome analysis of decidual Mϕ, Hofbauer cells, neonatal, and maternal blood monocytes (Mo)	Both decidual Mϕ and Hofbauer cells exhibited hypermethylation of genes encoding classical Mϕ activation	(25)
				Both decidual Mϕ and Hofbauer cells exhibited hypomethylation of genes encoding alternative Mϕ macrophage activation	

The role of phosphatidylserine receptor Tim-3 in macrophages from the maternal–fetal interface has gained recent attention (30). Tim-3 is recognized as a regulator of pro- and anti-inflammatory innate immune responses (31), and Tim-3 expression is enhanced in M2 immunoregulatory macrophages (32, 33). Therefore, Tim-3 may play an important role in maintaining macrophage mediated immune tolerance at the maternal–fetal interface (31). Indeed, Tim-3 facilitates phagocytic activity of apoptotic bodies at the maternal–fetal interface, and blockade of Tim-3 on uterine macrophages led to increased production of IFN-γ and TNF-α by inflammatory granulocytes with subsequent rejection of the fetus (30). To date, it is unknown if loss of Tim-3 or dysregulation of Tim-3 occurs in adverse pregnancy outcome.

Hofbauer cells are macrophages that reside within the mesenchymal stroma of the chorionic villi (34), which are thought to originate from fetal hematopoietic cells (35). In human placenta, Hofbauer cells have been detected by 4 weeks gestation, and their numbers increase during the first trimester and then decline by term (36). Based on their hypermethylation patterns, Hofbauer cells display a commitment to M2 in healthy pregnancy (25). Other phenotypic features that suggest these cells are pro-M2 include constitutive expression of DC-sign (CD209), high levels of CD163, CD68, CD45, hyaluronan receptor LYVE-1, and HLA antigens A, B, and C, IL-10, and TGF-β (37–39). Hofbauer cells may have a role in placental angiogenesis, remodeling of the extracellular matrix, and modulation of inflammation, which are M2 characteristics (39–41). Although not a particular feature of macrophage function, it has been suggested that Hofbauer cells participate in the regulation of stromal fluid balance, ion exchange, and transfer of serum proteins to the vascular system (36).

## Anatomy of the Maternal–Fetal Interface in Relation to Macrophages

The various compartments of the maternal–fetal interface (placenta, decidua, and myometrium) are anatomically distinguished by the composition of the connective tissue and the leukocyte populations present in each compartment. For example, macrophages residing in the myometrium may be receiving signals from T-cells, dendritic cells, and myocytes, whereas macrophages within the decidua receive signals from decidualized stromal cells, uterine NK cells, T-cells, and trophoblasts. In addition, decidual macrophages respond to immunoregulatory compounds such as HLA-G, TGF-β, and vasoactive intestinal peptide (VIP) that are produced by adjacent trophoblasts (40–42).

The mammalian uterus has two distinct tissue layers: the endometrium and myometrium. Macrophages are found interspersed in both layers of the uterus. The luminal surface of the endometrium consists of simple cuboidal epithelium that is supported by underlying connective tissue. The endometrium contains a complex vascular supply and a diverse population of leukocytes that changes with stage of the menstrual cycle and stage of pregnancy (43). The myometrium is composed of interlacing bundles of smooth muscle. In human beings, the myometrium exhibits minimal change during pregnancy (44). In rodents, however, the myometrium below the site of placental attachment expands and develops into the metrial triangle (44). Prior to embryo implantation, the endometrium begins to undergo structural changes referred to as decidualization, which is initiated by increased progesterone levels (44, 45). The process of decidualization in human beings stabilizes by 12 weeks gestation (46). The physical changes that occur during decidualization include proliferation of luminal epithelium, development of secretory glandules with large apical protrusions (pinopodes) and microvilli, and transformation of fibroblast-like endometrial stromal cells into larger rounded cells (47, 48). In addition, endometrial cells produce a variety of compounds that attract leukocytes to the decidualized tissue such as prolactin, colony stimulating factor 1 (CSF-1), macrophage inhibitory factor (MIF), IL-15, insulin growth factor binding protein-1 (IGFBP-1), and cyclooxygenase-2 (COX-2), and cell adhesion molecules (ICAM-1, VACM-1, LFA-3, H-CAM) (41, 45, 48, 49). Both CSF-1and MIF influence the recruitment of macrophages into the decidua (11, 43, 50, 51).

Following embryo implantation, the decidua differentiates into two distinct regions: the decidua basalis, which refers to the portion of the uterus attached to the placenta, and decidua parietalis, which refers to the rest of the endometrium lining the main cavity of the pregnant uterus. In human beings, macrophages within the decidua basalis coexist with trophoblasts, CD56^bright^/CD16^low^ NK cells, and T-cells, 25% of which are CD4^+^/CD25^+^/FOXP3^+^ regulatory T-cells (Tregs) (11, 44). Within the decidua basalis, CD14^+^ macrophages and NK cells are often observed in aggregates in association with spiral arteries (11, 52). Decidual macrophages are also observed in close association with trophoblasts (40). Factors released by neighboring trophoblasts may induce decidual trophoblasts to display cell surface expression of CD14 and CD16, exhibit increased phagocytic capacity, and secrete more IL-1β, IL-10, and IP-10 (53).

The microenvironment within the myometrium slightly differs from the decidual basalis in that macrophages and T-cells are the predominant leukocyte populations, and there are few CD56^bright^/CD16^low^ NK cells (54). In rats and mice, macrophages are more predominant in the myometrium than in the decidua, which has been attributed to higher CSF-1 concentrations within this compartment (55). Trophoblasts are also present within the inner third of the myometrium in both human beings and rats, but not in mice (14, 56). Macrophages recruited into the myometrium are important for the normal induction of labor and post-partum uterine remodeling (5, 57).

Placentation in human beings, non-human primates, and rodents is described as hemochorial because there are no barriers between trophoblast cells and maternal blood (58). Hemochorial placentation requires extensive tissue remodeling in which the endometrium that becomes the decidua basalis is obliterated and the uterine arteries are transformed from high resistance, low-flow arteries to low resistance, and high-flow vessels (14). During remodeling of spiral arteries, the endothelium and smooth muscle cells are replaced by invading extravillous trophoblasts and fibrinoid deposits (59). This complex process depends on a coordinated crosstalk between decidual stromal cells, uterine NK cells, and CD14^+^/CD68^+^ macrophages (59–62). Under optimal circumstances, the transient invasion of uterine NK cells and CD14^+^/CD68^+^ macrophages into the spiral arteries pave the way for the second stage of remodeling that involves invasion of trophoblasts into the vessels (52, 59).

## M1 or M2 in Normal Pregnancy, it is in the Timing

It has been recently proposed that pregnancy is actually an active and highly regulated immunologic process (63). Successful implantation requires a transient inflammatory phase that is initiated by cytokines and prostaglandins within seminal fluid (64). During the peri-implantation period, the polarization pattern of decidual macrophages is skewed toward M1 (65). However, as trophoblasts establish attachment into the endometrial lining and invade the uterine stroma, decidual macrophages begin to transition to a mixed M1/M2 profile (65). This mixed population may represent M1/M2 or possibly a blend of M2 subtypes (pro-inflammatory M2b mixed with anti-inflammatory M2a and M2c). This mixed polarization pattern continues through the first trimester and the early phase of the second trimester of pregnancy when the uterine vasculature is undergoing extensive remodeling in order to establish an adequate placental–fetal blood supply (63). After placental development is complete, the decidua shifts toward a pro-M2 environment, which prevents rejection of the fetus and allows fetal growth until parturition. Parturition, which is another inflammatory event, is preceded by an influx of macrophages into the myometrium and decidua (3–5). This inflammatory process promotes the contraction of the uterus, expulsion of the baby, ejection of the placenta, and uterine involution (57).

A2V, which is an isoform of the alpha subunit of vacuolar ATPase (V-ATPase), is a key immune regulator important for implantation (66). At least in normal murine pregnancy, A2V is expressed in sperm, embryo, and endometrium (65), and loss of a2v results in reproductive failure (67). In the reproductive tract, a2V promotes a transient pro-inflammatory effect followed by a balanced immune response that facilitates embryo implantation without rejection. During the pre-implantation period, cleavage of a2V in semen fluid releases a soluble N-terminus portion of the protein (a2NTD). Seminal a2NTD has pro-inflammatory effects such as upregulation of leukemia inhibitory factor (LIF), IL-1β, TNF-α, and MCP-1 gene expression in the uterus, creating a transient pro-M1 effect. The developing embryo continues to produce a2V, which continues to stimulate uterine MCP-1. This in turn attracts new macrophages into the endometrium leading to a more balanced M1/M2 ratio and protection against rejection of the fetus. In the BALB/c mouse, these studies clearly demonstrate that a2V mediated induction of MCP-1 is important for proper regulation of M1/M2 during early pregnancy (65, 67). Additional factors present within the decidualized tissue that influence macrophage polarization of invading macrophages also contribute to tolerance.

As pregnancy progresses, the M1/M2 ratio decreases (23, 27) and the population of decidual macrophages become more heterogeneous. This, in part, is caused by the uneven spatial distribution of neighboring leukocytes and trophoblasts within the decidua, which provide different cues to resident macrophages. Events that account for these changes include the contribution of immunoregulatory molecules from trophoblasts such as HLA-G (68, 69), TGF-β (40), and VIP (41). These compounds have direct effects on macrophages or on Tregs, which express pro-M2 factors such as IDO and IL-10 (1). In contrast, CD56^bright^/CD16^low^ NK cells, which are intimately associated with decidual macrophages invading the spiral arteries, produce pro-M1 IFN-γ (70) and TNF-α (11). Houser et al. identified two distinct macrophage subsets in first trimester decidua based on the degree of CD11c expression (24). Regardless of the degree of Cd11c expression, these cells uniformly expressed CD68 and CD14. CD11c^HI^ macrophages expressed less phagocytic receptors such as CD209 (DC-SIGN) and CD206 (mannose receptor) compared to CD11c^LO^ macrophages. CD11c^HI^ and CD11c^LO^ macrophages also had different transcriptional profiles. For example, CD11c^HI^ macrophages express genes involved in invasion, mobility, inflammatory processes including lipid metabolism, and anti-apoptotic effects, whereas CD11c^LO^ macrophages express genes that regulate growth and development, as well extracellular communication including networking. CD11c^LO^ macrophages may be the subpopulation of cells that actively suppress CD56^bright^/CD16^low^ NK cell mediated killing of invading trophoblasts (2). On the other hand, the transcriptional profile of CD11c^HI^ macrophages suggests that these cells participate in the remodeling of the uterine arteries that occurs during this stage of pregnancy.

During the growth phase of pregnancy, the predominant immunological feature is the induction of an anti-inflammatory state. It is presumed that the immunosuppressive qualities observed in decidual macrophages studied from first trimester pregnancies continue to be present as pregnancy advances (71). This notion is supported by a several studies that evaluated macrophages isolated from term decidua. First, the methylation pattern of these cells show silencing of genes that encode M1 markers such as TLR9, IL1B, IL12RB2, CD48, and FGR, whereas genes associated with M2 polarity such as CCL13, CCL14, A2M, HNMT, and IL10 were hypomethylated (25). Second, decidual macrophages express M2 polarity characteristics such as reduced expression of co-stimulatory molecule CD86 with spontaneous production of high levels of IL-10 and IDO (72). Third, decidual macrophages express C-type lectin receptors such as CD206 throughout pregnancy (73), which is more in line with an M2 phenotype (71).

Normal parturition is an inflammatory process characterized by an enhanced expression of IL-1β, IL-6, and IL-8 with an accumulation of leukocytes in the cervix, fetal membranes, decidua, and myometrium (74). Macrophages are one of the predominant cell types in the decidua and myometrium during normal parturition. CD68^+^ macrophages begin to invade the decidua a few days before parturition (3, 4), which coincides with a global increase in the production of chemokines such as CCL2 (MCP-1), CCL4 (MIP-1β), CCL5 (Rantes), CXCL8 (IL-8), and CXCL10 (IP-10) within the decidua (75). Immediately before parturition, a wave of macrophages invades the myometrium (4), resulting in an increase in pro and anti-inflammatory cytokines such as IL-1β, IL-6, IL-12, and IL-10. To date, the polarity profile of macrophages invading the maternal–fetal interface before or during labor has not been determined.

Although the density of Hofbauer cells within the villous mesenchyme fluctuates with gestational age (36), the limited characterization of these cells suggests that they display a commitment to M2 related functions. DNA methylation profiling of Hofbauer cells obtained from term placentas show that pro-M1 genes such as TLR9, IL1B, IL12RB2, CD48, and FGR are silenced (25). In contrast, pro-M2 genes such as CCL2, CCL13, CCL14, CD209, and A2M are hypomethylated, and thus, available for expression (25). Hofbauer cells constitutively express anti-inflammatory cytokines such as IL-10 and TGF-β1 (38), which may in part be related to the immune suppressive effects of neighboring mesenchymal stem cells that have the capacity to shift macrophages from an M1 phenotype to M2 (76). It is also likely that arginase I and II activity from surrounding villous trophoblasts (22) reduces extracellular arginine pools within the villous mesenchyme that would be required for translation of iNOS and differentiation into M1.

## M1/M2 Imbalances during Early Pregnancy

It is noteworthy that some obstetric complications associated with decidual M1/M2 imbalances occur during the early inflammatory phase of pregnancy. Under these circumstances, it is likely that the surrounding environment supports M1 polarization over M2. Some examples include spontaneous abortions and disorders involving inadequate remodeling of the uterine arteries.

Spontaneous abortions that occur within 12 weeks gestation are associated with an increased influx of macrophages in the decidual stroma (12, 77). In one study, decidual macrophages from spontaneous abortions had increased Fas-L expression, which coincided with an increased rate of trophoblast apoptosis (12). It was proposed that the increased FasL-expressing population of macrophages reflected M1 activation (12); however, this is yet to be proven. Jaiswal et al. recently demonstrated that a shift toward M1 polarization at the maternal–fetal interface enhances abortion in CBA × DBA/2 mouse matings treated with LPS (67). The underlying mechanism involved a significant decrease in placental a2V coupled with a decrease in uterine MCP-1 expression.

Inadequate remodeling of the uterine spiral arteries is associated with a spectrum of obstetric complications such as pre-eclampsia, intrauterine growth restriction, pre-term birth, pre-term premature rupture of membranes (PPROM), late sporadic miscarriage, and premature separation of the placenta from the uterus (78). During optimal spiral artery remodeling, there is a transient influx of NK cells and activated CD68^+^ macrophages into the vessel wall. This leukocytic influx, which is described as stage 1 remodeling, is thought to initiate the disruption of the organized smooth muscle layer and endothelium needed for subsequent trophoblast invasion into the vessel (59). Pro-M2 stromal macrophages in the outer periphery of the vessel assist in controlling inflammation by phagocytizing potentially pro-inflammatory cellular debris and secreting anti-inflammatory TGF-β1 (40). Excessive M1 or M2b activity during the early phase of remodeling can prevent the resolution of this inflammatory phase, thus, disrupting trophoblast invasion into the arterial wall and preventing complete remodeling of the vessel (52, 59). Indeed, reduced trophoblast invasion into utero-placental spiral arteries is associated with an excess of activated macrophages in and around these arteries (79, 80). Macrophages activated by exogenous TNF-α, tryptophan depletion (which reduces production of IDO), GM-CSF, or M-CSF promote apoptosis of extravillous trophoblasts *in vitro* (80, 81).

## M1/M2 Imbalances during Late Pregnancy

Decidual inflammation is also presumed to produce M1 or M2b excess in the pathogenesis of uterine atherosis. Uterine atherosis is a late pregnancy lesion of the decidua that is characterized by the accumulation of lipid filled CD68^+^ foamy macrophages in the subendothelial layer of uterine spiral arteries (14, 82). This lesion is also associated with increased local TNF-α (83). In rare instances, acute atherosis has also been observed in the myometrial segments of the spiral arteries (78, 82). During early pregnancy, acute atherosis can also be seen in cases of defective spiral artery remodeling (78). In this instance, the lesions are present in the lower section of the arteries where they feed into the intervillous space (82).

Staff and Redman proposed that acute atherosis is an end result of various inflammatory pathways triggered by immunologic, genetic, and/or hemodynamic influences (sequela to impaired spiral arterial remodeling and perturbed laminar blood flow) that may be working singly or in combination (82). This hypothesis is based on the observation that acute atherosis is found in a wide range of pregnancy complications such as pre-eclampsia, fetal growth restriction, and certain autoimmune diseases (systemic lupus erythematosus and antiphospholipid syndrome). It has also been suggested that infectious organisms implicated in promoting cardiovascular disease such as *Chlamydia pneumoniae* may play a role in initiating or activating uterine atherosis (84). Recent work in our laboratory suggests that the periodontal pathogen, *Porphyromonas gingivalis*, which is implicated in promoting cardiovascular disease (85), may indeed have such an effect.

## M1/M2 Imbalances Associated with Intrauterine Infection

*Porphyromonas gingivalis* is a common periodontal pathogen of human beings that is also implicated in low-birth weight, fetal growth restriction, pre-eclampsia, and spontaneous pre-term birth (86–93). In prototypical M2 Balb/c mice, intrauterine infection with *P. gingivalis* induces pro-M1 or M2b inflammatory responses such as increased TNF-α and IFN-γ with suppression of IL-10, and fetal growth restriction (94, 95). Using a rat model of intrauterine infection, we observed that the local presence of *P. gingivalis* within the uterus produced lesions suggestive of an M1 > M2 or M2b > M2a, M2c imbalance. Namely, infected dams exhibited acute arteritis within the endometrium and metrial triangle characterized by perivascular necrosis, hyaline degeneration with varying degrees of thrombosis (96, 97). Moreover, the spiral arteries had increased densities of CD68^+^ macrophages, with increased stromal TNF-α and a concomitant decrease in extravillous trophoblast invasion into the placental bed. Since this was an acute study (4 days duration), we did not observe lipid filled macrophages surrounding affected spiral arteries. Nevertheless, these results provide a compelling argument that certain bacterial infections may promote M1/M2 imbalances that in the placental bed have a negative impact on spiral artery remodeling.

To date, there is no evidence that pro-M1 activity is actually involved in the pathogenesis of chorioamnionitis and pre-term delivery. Rather, acute chorioamnionitis may actually promote M2 polarization. Decidual macrophages in gestation day 16 FVB/NJ mice sustain an M2 polarity phenotype even after LPS treatment (98). C57BL/6 mice, which are resistant to chorioamnionitis during intrauterine infection with *Ureaplasma parvum* (99), do not develop pro-M1 responses, instead C57BL/6 mice exhibit a pro-M2 profile within the placenta and decidua (100). This is particularly intriguing since C57BL/6 mice are known for their prototypical M1 immune responses (8). Human chorioamniotic and umbilical cord macrophages express more nuclear IL-33 (also known as full length IL-33) during acute chorioamnionitis (101). Nuclear IL-33 acts as a transcriptional regulator that has been shown to suppress production of LPS-stimulated pro-inflammatory cytokines *in vitro* (102). Moreover, extracellular or mature IL-33 released from activated or dying cells promotes M2 polarization of macrophages at the site of inflammation (103). At best, pro-M2 activation during chorioamnionitis may minimize detrimental inflammation associated with chorioamnionitis, or it may enhance infection by suppressing antimicrobial responses.

Manipulation of macrophage polarity at the maternal–fetal interface by infectious agents can impact pregnancy outcomes. The obligate intracellular parasite *Toxoplasma gondii* can invade the placenta and fetus, resulting in spontaneous abortion, stillbirth, fetal neurological, and ocular damage (104). A virulent strain of *T. gondii*, TgCtwh3, actively induces M2 polarization in infected macrophages (15). TgCtwh3 rapidly invades the placenta producing a heavy parasite burden with minimal inflammation but a high rate of trophoblast apoptosis (15). On the other hand, less virulent strain TgCtwh6 triggers pro-M1 responses in infected macrophages (15). TgCtwh6 can still invade the placenta, but infection is contained and associated with a profound lymphocytic inflammatory response with trophoblast apoptosis that is less extensive than TgCtwh3 (15).

## Hofbauer Cells in Complicated Pregnancy

Placental Hofbauer cells are responsive to paracrine signals from surrounding cells within the chorionic villus (105). Alterations in Hofbauer cell densities within the villi have been reported during chorioamnionitis (106, 107), but there is no evidence that these cells shift to M1 polarity phenotype during chorioamnionitis. Instead, HIV infected Hofbauer cells or cells from chorioamnionitis cases continue to display M2 characteristics such as production of IL-10, TGF-β, expression of DC-SIGN, CD163, and mannose receptor/CD206 (38, 39). In another study, Hofbauer cells from chorioamnionitis patients and healthy controls had similar gene expression profiles, which were not considered to be committed to either M1 or M2 (108).

Interestingly, it was reported that hyperglycemia can shift Hofbauer cells toward M1 activation (109). Hofbauer cells isolated from diabetic women displayed characteristics that could be attributed to either M1or M2b phenotype (decreased CD163, CD209, IL-10 with increased CD68, CCR7, and IL-1β) (109). Further, when rat Hofbauer cells were cultured in high-glucose conditions *in vitro*, these cells expressed increased levels of NOS2 gene expression and NO, clear markers of M1 activation (109). But a caveat of that experiment was that Hofbauer cells were cultured in RPMI media, which contains supraphysiologic levels of arginine (7). High-arginine concentrations in RPMI are not representative of the tissue microenvironment, especially during inflammation (7). Further, high-arginine levels will promote iNOS activation in macrophages *in vitro* (7).

Folate receptor β (FR-β) is preferentially expressed on M2 polarized macrophages, and is considered a biomarker for immunoregulatory M2 macrophages (110). Decreased expression of FR-β and CD163, but not CD68, has been observed in Hofbauer cells isolated from women with severe pre-term pre-eclampsia (111) suggesting that in this syndrome, Hofbauer cells may have shifted toward M1 polarity. This would be consistent with Aziza et al.’s study (112) that reported increased iNOS with concurrent decreased eNOS in placentas from women with pre-eclampsia.

Villitis of unknown etiology (VUE) is an inflammatory lesion of the chorionic villi that is associated with intrauterine fetal growth restriction and perinatal morbidity and mortality (105). VUE is characterized by influxes of Hofbauer cells, maternal T-cells (more CD8^+^ than CD4^+^), and increased expression of chemokines (CXCL9, CXCL10, CXCL11, CXCL13, CCL4, CCL5, CXCR3, CCR5) within the placental villi (113, 114). It has been suggested that the increased expression of chemokines and their receptors within the villous mesenchyme of VUE patients provides the pro-migratory signals that promote increased migration of Hofbauer cells and maternal T-cells into the villous (115). Hofbauer cells do migrate in response to fibroblast secreted MCP-1 *in vitro* (106). Whether or not changes in villous mesenchyme promote M1 polarization of Hofbauer cells remains to be determined.

## Conclusion

Macrophages serve important roles in the development of hemochorial placentation, maintenance of pregnancy, and initiation of parturition. As cells that are highly responsive to altering their polarization pattern *in vivo*, the mixed M polarity phenotypes present in the decidua reflect the heterogeneous nature of the maternal–fetal interface. Both spatial and temporal regulation of M1 and M2 polarization is required for successful pregnancy. Similarly, ill-timed or ill-placed macrophage polarization at the maternal–fetal interface is associated with pregnancy complications and poor outcomes. Despite recent insights into M1/M2 dynamics at the maternal–fetal interface, there are still critical knowledge gaps concerning the *in situ* context of macrophage polarity, the mechanisms that promote dysregulation, and its impact on healthy pregnancy and obstetric disease.

## Conflict of Interest Statement

The authors declare that the research was conducted in the absence of any commercial or financial relationships that could be construed as a potential conflict of interest.

## References

[1] Nagamatsu T Schust DJ. The immunomodulatory roles of macrophages at the maternal-fetal interface. Reprod Sci (2010) 17(3):209–18. 10.1177/1933719109349962 20065301

[2] Co EC Gormley M Kapidzic M Rosen DB Scott MA Stolp HA Maternal decidual macrophages inhibit NK cell killing of invasive cytotrophoblasts during human pregnancy. Biol Reprod (2013) 88(6):155. 10.1095/biolreprod.112.099465 23553431 PMC4070869

[3] Hamilton S Oomomian Y Stephen G Shynlova O Tower CL Garrod A Macrophages infiltrate the human and rat decidua during term and preterm labor: evidence that decidual inflammation precedes labor. Biol Reprod (2012) 86(2):39. 10.1095/biolreprod.111.095505 22011391

[4] Shynlova O Nedd-Roderique T Li Y Dorogin A Nguyen T Lye SJ. Infiltration of myeloid cells into decidua is a critical early event in the labour cascade and post-partum uterine remodelling. J Cell Mol Med (2013) 17(2):311–24. 10.1111/jcmm.12012 23379349 PMC3822594

[5] Shynlova O Nedd-Roderique T Li Y Dorogin A Lye SJ. Myometrial immune cells contribute to term parturition, preterm labour and post-partum involution in mice. J Cell Mol Med (2013) 17(1):90–102. 10.1111/j.1582-4934.2012.01650.x 23205502 PMC3823139

[6] Mantovani A Sica A Sozzani S Allavena P Vecchi A Locati M. The chemokine system in diverse forms of macrophage activation and polarization. Trends Immunol (2004) 25(12):677–86. 10.1016/j.it.2004.09.015 15530839

[7] Mills CD. Macrophage arginine metabolism to ornithine/urea or nitric oxide/citrulline: a life or death issue. Crit Rev Immunol (2001) 21(5):399–425. 10.1615/CritRevImmunol.v21.i5.10 11942557

[8] Mills CD Kincaid K Alt JM Heilman MJ Hill AM. M-1/M-2 macrophages and the Th1/Th2 paradigm. J Immunol (2000) 164(12):6166–73. 10.4049/jimmunol.164.12.6166 10843666

[9] Mills CD. M1 and M2 macrophages: oracles of health and disease. Crit Rev Immunol (2012) 32(6):463–88. 10.1615/CritRevImmunol.v32.i6.10 23428224

[10] Comalada M Yeramian A Modolell M Lloberas J Celada A. Arginine and macrophage activation. Methods Mol Biol (2012) 844:223–35. 10.1007/978-1-61779-527-5_16 22262446

[11] Erlebacher A. Immunology of the maternal-fetal interface. Annu Rev Immunol (2013) 31:387–411. 10.1146/annurev-immunol-032712-100003 23298207

[12] Guenther S Vrekoussis T Heublein S Bayer B Anz D Knabl J Decidual macrophages are significantly increased in spontaneous miscarriages and over-express FasL: a potential role for macrophages in trophoblast apoptosis. Int J Mol Sci (2012) 13(7):9069–80. 10.3390/ijms13079069 22942752 PMC3430283

[13] Wang WJ Hao CF Lin QD. Dysregulation of macrophage activation by decidual regulatory T cells in unexplained recurrent miscarriage patients. J Reprod Immunol (2011) 92(1–2):97–102. 10.1016/j.jri.2011.08.004 22015003

[14] Pijnenborg R Vercruysse L Hanssens M. The uterine spiral arteries in human pregnancy: facts and controversies. Placenta (2006) 27(9–10):939–58. 10.1016/j.placenta.2005.12.006 16490251

[15] Liu T Zhang Q Liu L Xu X Chen H Wang H Trophoblast apoptosis through polarization of macrophages induced by Chinese *Toxoplasma gondii* isolates with different virulence in pregnant mice. Parasitol Res (2013) 112(8):3019–27. 10.1007/s00436-013-3475-3 23722717

[16] Martinez FO Gordon S. The M1 and M2 paradigm of macrophage activation: time for reassessment. F1000Prime Rep (2014) 6:13. 10.12703/P6-13 24669294 PMC3944738

[17] Mosser DM Edwards JP Exploring the full spectrum of macrophage activation. Nat Rev Immunol (2008) 8(12):958–69 10.1038/nri2448 19029990 PMC2724991

[18] Kurowska-Stolarska M Stolarski B Kewin P Murphy G Corrigan CJ Ying S IL-33 amplifies the polarization of alternatively activated macrophages that contribute to airway inflammation. J Immunol (2009) 183(10):6469–77. 10.4049/jimmunol.0901575 19841166

[19] Nelson MP Christmann BS Werner JL Metz AE Trevor JL Lowell CA IL-33 and M2a alveolar macrophages promote lung defense against the atypical fungal pathogen *Pneumocystis murina*. J Immunol (2011) 186(4):2372–81 10.4049/jimmunol.1002558 21220696 PMC4274743

[20] Pesce JT Ramalingam TR Mentink-Kane MM Wilson MS El Kasmi KC Smith AM Arginase-1-expressing macrophages suppress Th2 cytokine-driven inflammation and fibrosis. PLoS Pathog (2009) 5(4):e1000371. 10.1371/journal.ppat.1000371 19360123 PMC2660425

[21] El-Gayar S Thuring-Nahler H Pfeilschifter J Rollinghoff M Bogdan C. Translational control of inducible nitric oxide synthase by IL-13 and arginine availability in inflammatory macrophages. J Immunol (2003) 171(9):4561–8. 10.4049/jimmunol.171.9.4561 14568929

[22] Ishikawa T Harada T Koi H Kubota T Azuma H Aso T. Identification of arginase in human placental villi. Placenta (2007) 28(2–3):133–8. 10.1016/j.placenta.2006.03.015 16720041

[23] Gustafsson C Mjosberg J Matussek A Geffers R Matthiesen L Berg G Gene expression profiling of human decidual macrophages: evidence for immunosuppressive phenotype. PLoS One (2008) 3(4):e2078. 10.1371/journal.pone.0002078 18446208 PMC2323105

[24] Houser BL Tilburgs T Hill J Nicotra ML Strominger JL. Two unique human decidual macrophage populations. J Immunol (2011) 186(4):2633–42. 10.4049/jimmunol.1003153 21257965 PMC3712354

[25] Kim SY Romero R Tarca AL Bhatti G Kim CJ Lee J Methylome of fetal and maternal monocytes and macrophages at the feto-maternal interface. Am J Reprod Immunol (2012) 68(1):8–27. 10.1111/j.1600-0897.2012.01108.x 22385097 PMC3479407

[26] Repnik U Tilburgs T Roelen DL van der Mast BJ Kanhai HH Scherjon S Comparison of macrophage phenotype between decidua basalis and decidua parietalis by flow cytometry. Placenta (2008) 29(5):405–12. 10.1016/j.placenta.2008.02.004 18353434

[27] Svensson J Jenmalm MC Matussek A Geffers R Berg G Ernerudh J. Macrophages at the fetal-maternal interface express markers of alternative activation and are induced by M-CSF and IL-10. J Immunol (2011) 187(7):3671–82. 10.4049/jimmunol.1100130 21890660

[28] Laskarin G Medancic SS Redzovic A Duric D Rukavina D. Specific decidual CD14(+) cells hamper cognate NK cell proliferation and cytolytic mediator expression after mucin 1 treatment in vitro. J Reprod Immunol (2012) 95(1–2):36–45. 10.1016/j.jri.2012.06.002 22841164

[29] Laskarin G Cupurdija K Tokmadzic VS Dorcic D Dupor J Juretic K The presence of functional mannose receptor on macrophages at the maternal-fetal interface. Hum Reprod (2005) 20(4):1057–66. 10.1093/humrep/deh740 15746201

[30] Chabtini L Mfarrej B Mounayar M Zhu B Batal I Dakle PJ TIM-3 regulates innate immune cells to induce fetomaternal tolerance. J Immunol (2013) 190(1):88–96. 10.4049/jimmunol.1202176 23180822 PMC3529820

[31] Han G Chen G Shen B Li Y. Tim-3: an activation marker and activation limiter of innate immune cells. Front Immunol (2013) 4:449. 10.3389/fimmu.2013.00449 24339828 PMC3857553

[32] Zhang ZY Schluesener HJ Zhang Z. Distinct expression of Tim-3 during different stages of rat experimental autoimmune neuritis. Brain Res Bull (2011) 86(3–4):229–34. 10.1016/j.brainresbull.2011.07.005 21784136

[33] Frisancho-Kiss S Nyland JF Davis SE Barrett MA Gatewood SJ Njoku DB Cutting edge: T cell Ig mucin-3 reduces inflammatory heart disease by increasing CTLA-4 during innate immunity. J Immunol (2006) 176(11):6411–5. 10.4049/jimmunol.176.11.6411 16709797

[34] Takahashi K Naito M Katabuchi H Higashi K. Development, differentiation, and maturation of macrophages in the chorionic villi of mouse placenta with special reference to the origin of Hofbauer cells. J Leukoc Biol (1991) 50(1):57–68. 2056247 10.1002/jlb.50.1.57

[35] Karakaya YA Ozer E. The role of Hofbauer cells on the pathogenesis of early pregnancy loss. Placenta (2013) 34(12):1211–5. 10.1016/j.placenta.2013.10.010 24199671

[36] Ingman K Cookson VJ Jones CJ Aplin JD. Characterisation of Hofbauer cells in first and second trimester placenta: incidence, phenotype, survival in vitro and motility. Placenta (2010) 31(6):535–44. 10.1016/j.placenta.2010.03.003 20347485

[37] Bockle BC Solder E Kind S Romani N Sepp NT. DC-sign+ CD163+ macrophages expressing hyaluronan receptor LYVE-1 are located within chorion villi of the placenta. Placenta (2008) 29(2):187–92. 10.1016/j.placenta.2007.11.003 18078989

[38] Johnson EL Chakraborty R. Placental Hofbauer cells limit HIV-1 replication and potentially offset mother to child transmission (MTCT) by induction of immunoregulatory cytokines. Retrovirology (2012) 9:101. 10.1186/1742-4690-9-101 23217137 PMC3524025

[39] Joerink M Rindsjo E van Riel B Alm J Papadogiannakis N. Placental macrophage (Hofbauer cell) polarization is independent of maternal allergen-sensitization and presence of chorioamnionitis. Placenta (2011) 32(5):380–5. 10.1016/j.placenta.2011.02.003 21419483

[40] Abrahams VM Kim YM Straszewski SL Romero R Mor G. Macrophages and apoptotic cell clearance during pregnancy. Am J Reprod Immunol (2004) 51(4):275–82. 10.1111/j.1600-0897.2004.00156.x 15212680

[41] Grasso E Paparini D Aguero M Mor G Perez Leiros C Ramhorst R. VIP contribution to the decidualization program: regulatory T cell recruitment. J Endocrinol (2014) 221(1):121–31. 10.1530/JOE-13-0565 24492467

[42] Hunt JS Petroff MG Burnett TG. Uterine leukocytes: key players in pregnancy. Semin Cell Dev Biol (2000) 11(2):127–37. 10.1006/scdb.2000.0158 10873709

[43] Thiruchelvam U Dransfield I Saunders PT Critchley HO. The importance of the macrophage within the human endometrium. J Leukoc Biol (2013) 93(2):217–25. 10.1189/jlb.0712327 23108100

[44] Hunt JS Pollard JW Macrophages in the uterus and placenta. Curr Top Microbiol Immunol (1992) 181:39–63. 1424785 10.1007/978-3-642-77377-8_2

[45] Kammerer U von Wolff M Markert UR Immunology of human endometrium. Immunobiology (2004) 209(7):569–74 10.1016/j.imbio.2004.04.009 15568621

[46] Svensson-Arvelund J Ernerudh J Buse E Cline JM Haeger JD Dixon D The placenta in toxicology. Part II: systemic and local immune adaptations in pregnancy. Toxicol Pathol (2014) 42(2):327–38. 10.1177/0192623313482205 23531796

[47] Paria BC Reese J Das SK Dey SK. Deciphering the cross-talk of implantation: advances and challenges. Science (2002) 296(5576):2185–8. 10.1126/science.1071601 12077405

[48] Dunn CL Kelly RW Critchley HO. Decidualization of the human endometrial stromal cell: an enigmatic transformation. Reprod Biomed Online (2003) 7(2):151–61. 10.1016/S1472-6483(10)61745-2 14567882

[49] Strakova Z Srisuparp S Fazleabas AT. IL-1beta during in vitro decidualization in primate. J Reprod Immunol (2002) 55(1–2):35–47. 10.1016/S0165-0378(01)00141-3 12062820

[50] Bulmer JN Pace D Ritson A. Immunoregulatory cells in human decidua: morphology, immunohistochemistry and function. Reprod Nutr Dev (1988) 28(6B):1599–613. 10.1051/rnd:19881006 3073448

[51] Arcuri F Buchwalder L Toti P Cintorino M Tosi P Lockwood CJ Differential regulation of colony stimulating factor 1 and macrophage migration inhibitory factor expression by inflammatory cytokines in term human decidua: implications for macrophage trafficking at the fetal-maternal interface. Biol Reprod (2007) 76(3):433–9. 10.1095/biolreprod.106.054189 17108334

[52] Harris LK. IFPA Gabor than Award Lecture: transformation of the spiral arteries in human pregnancy: key events in the remodelling timeline. Placenta (2011) 32(Suppl 2):S154–8. 10.1016/j.placenta.2010.11.018 21167598

[53] Aldo PB Racicot K Craviero V Guller S Romero R Mor G. Trophoblast induces monocyte differentiation into CD14+/CD16+ macrophages. Am J Reprod Immunol (2014) 72(3):270–84. 10.1111/aji.12288 24995492 PMC4230492

[54] Ivanisevic M Segerer S Rieger L Kapp M Dietl J Kammerer U Antigen-presenting cells in pregnant and non-pregnant human myometrium. Am J Reprod Immunol (2010) 64(3):188–96. 10.1111/j.1600-0897.2010.00858.x 20528834

[55] Tagliani E Shi C Nancy P Tay CS Pamer EG Erlebacher A. Coordinate regulation of tissue macrophage and dendritic cell population dynamics by CSF-1. J Exp Med (2011) 208(9):1901–16. 10.1084/jem.20110866 21825019 PMC3171096

[56] Rosario GX Ain R Konno T Soares MJ. Intrauterine fate of invasive trophoblast cells. Placenta (2009) 30(5):457–63. 10.1016/j.placenta.2009.02.008 19344949 PMC2674526

[57] Shynlova O Tsui P Dorogin A Lye SJ. Monocyte chemoattractant protein-1 (CCL-2) integrates mechanical and endocrine signals that mediate term and preterm labor. J Immunol (2008) 181(2):1470–9. 10.4049/jimmunol.181.2.1470 18606702

[58] Benirschke K Pathology of the Human Placenta. New York, NY: Springer (2012).

[59] Harris LK. Review: trophoblast-vascular cell interactions in early pregnancy: how to remodel a vessel. Placenta (2010) 31(Suppl):S93–8. 10.1016/j.placenta.2009.12.012 20060584

[60] Smith SD Dunk CE Aplin JD Harris LK Jones RL. Evidence for immune cell involvement in decidual spiral arteriole remodeling in early human pregnancy. Am J Pathol (2009) 174(5):1959–71. 10.2353/ajpath.2009.080995 19349361 PMC2671283

[61] Bulmer JN Burton GJ Collins S Cotechini T Crocker IP Croy BA IFPA meeting 2011 workshop report II: angiogenic signaling and regulation of fetal endothelial function; placental and fetal circulation and growth; spiral artery remodeling. Placenta (2012) 33(Suppl):S9–14. 10.1016/j.placenta.2011.11.014 22177322

[62] Hazan AD Smith SD Jones RL Whittle W Lye SJ Dunk CE. Vascular-leukocyte interactions: mechanisms of human decidual spiral artery remodeling in vitro. Am J Pathol (2010) 177(2):1017–30. 10.2353/ajpath.2010.091105 20558572 PMC2913364

[63] Mor G Cardenas I Abrahams V Guller S. Inflammation and pregnancy: the role of the immune system at the implantation site. Ann N Y Acad Sci (2011) 1221:80–7. 10.1111/j.1749-6632.2010.05938.x 21401634 PMC3078586

[64] Robertson SA. Seminal fluid signaling in the female reproductive tract: lessons from rodents and pigs. J Anim Sci (2007) 85(13 Suppl):E36–44. 10.2527/jas.2006-578 17085725

[65] Jaiswal MK Mallers TM Larsen B Kwak-Kim J Chaouat G Gilman-Sachs A V-ATPase upregulation during early pregnancy: a possible link to establishment of an inflammatory response during preimplantation period of pregnancy. Reproduction (2012) 143(5):713–25. 10.1530/REP-12-0036 22454532

[66] Ntrivalas E Levine R Kwong C Gilman-Sachs A Beaman K. The a2 isoform of vacuolar ATPase is a modulator of implantation and feto-maternal immune tolerance in early pregnancy. J Reprod Immunol (2010) 85(1):106–11. 10.1016/j.jri.2009.10.010 20036779

[67] Jaiswal MK Gilman-Sachs A Chaouat G Beaman KD. Placental ATPase expression is a link between multiple causes of spontaneous abortion in mice. Biol Reprod (2011) 85(3):626–34. 10.1095/biolreprod.111.092494 21593477

[68] Lombardelli L Aguerre-Girr M Logiodice F Kullolli O Casart Y Polgar B HLA-G5 induces IL-4 secretion critical for successful pregnancy through differential expression of ILT2 receptor on decidual CD4(+) T cells and macrophages. J Immunol (2013) 191(7):3651–62. 10.4049/jimmunol.1300567 23997222

[69] Apps R Gardner L Sharkey AM Holmes N Moffett A. A homodimeric complex of HLA-G on normal trophoblast cells modulates antigen-presenting cells via LILRB1. Eur J Immunol (2007) 37(7):1924–37. 10.1002/eji.200737089 17549736 PMC2699429

[70] Fu B Li X Sun R Tong X Ling B Tian Z Natural killer cells promote immune tolerance by regulating inflammatory TH17 cells at the human maternal-fetal interface. Proc Natl Acad Sci U S A (2013) 110(3):E231–40. 10.1073/pnas.1206322110 23271808 PMC3549088

[71] Nagamatsu T Schust DJ. The contribution of macrophages to normal and pathological pregnancies. Am J Reprod Immunol (2010) 63(6):460–71. 10.1111/j.1600-0897.2010.00813.x 20163399

[72] Heikkinen J Mottonen M Komi J Alanen A Lassila O Phenotypic characterization of human decidual macrophages. Clin Exp Immunol (2003) 131(3):498–505 10.1046/j.1365-2249.2003.02092.x 12605704 PMC1808648

[73] Mues B Langer D Zwadlo G Sorg C Phenotypic characterization of macrophages in human term placenta. Immunology (1989) 67(3):303–7. 2788125 PMC1385344

[74] Osman I Young A Ledingham MA Thomson AJ Jordan F Greer IA Leukocyte density and pro-inflammatory cytokine expression in human fetal membranes, decidua, cervix and myometrium before and during labour at term. Mol Hum Reprod (2003) 9(1):41–5. 10.1093/molehr/gag001 12529419

[75] Hamilton SA Tower CL Jones RL. Identification of chemokines associated with the recruitment of decidual leukocytes in human labour: potential novel targets for preterm labour. PLoS One (2013) 8(2):e56946. 10.1371/journal.pone.0056946 23451115 PMC3579936

[76] Abumaree MH Al Jumah MA Kalionis B Jawdat D Al Khaldi A Abomaray FM Human placental mesenchymal stem cells (pMSCs) play a role as immune suppressive cells by shifting macrophage differentiation from inflammatory M1 to anti-inflammatory M2 macrophages. Stem Cell Rev (2013) 9(5):620–41. 10.1007/s12015-013-9455-2 23812784

[77] Lambropoulou M Tamiolakis D Venizelos J Liberis V Galazios G Tsikouras P Imbalance of mononuclear cell infiltrates in the placental tissue from foetuses after spontaneous abortion versus therapeutic termination from 8th to 12th weeks of gestational age. Clin Exp Med (2006) 6(4):171–6. 10.1007/s10238-006-0111-x 17191109

[78] Khong Y Brosens I Defective deep placentation. Best Pract Res Clin Obstet Gynaecol (2011) 25(3):301–11 10.1016/j.bpobgyn.2010.10.012 21109492

[79] Reister F Frank HG Heyl W Kosanke G Huppertz B Schroder W The distribution of macrophages in spiral arteries of the placental bed in pre-eclampsia differs from that in healthy patients. Placenta (1999) 20(2–3):229–33. 10.1053/plac.1998.0373 10195746

[80] Reister F Frank HG Kingdom JC Heyl W Kaufmann P Rath W Macrophage-induced apoptosis limits endovascular trophoblast invasion in the uterine wall of preeclamptic women. Lab Invest (2001) 81(8):1143–52. 10.1038/labinvest.3780326 11502865

[81] Wu ZM Yang H Li M Yeh CC Schatz F Lockwood CJ Pro-inflammatory cytokine-stimulated first trimester decidual cells enhance macrophage-induced apoptosis of extravillous trophoblasts. Placenta (2012) 33(3):188–94. 10.1016/j.placenta.2011.12.007 22212249 PMC3406179

[82] Staff AC Redman CW. IFPA award in placentology lecture: preeclampsia, the decidual battleground and future maternal cardiovascular disease. Placenta (2014) 35(Suppl):S26–31. 10.1016/j.placenta.2013.12.003 24411701

[83] Pijnenborg R McLaughlin PJ Vercruysse L Hanssens M Johnson PM Keith JC Jr Immunolocalization of tumour necrosis factor-alpha (TNF-alpha) in the placental bed of normotensive and hypertensive human pregnancies. Placenta (1998) 19(4):231–9. 10.1016/S0143-4004(98)90054-6 9639318

[84] Staff AC Dechend R Pijnenborg R. Learning from the placenta: acute atherosis and vascular remodeling in preeclampsia-novel aspects for atherosclerosis and future cardiovascular health. Hypertension (2010) 56(6):1026–34. 10.1161/HYPERTENSIONAHA.110.157743 20956732

[85] Reyes L Herrera D Kozarov E Rolda S Progulske-Fox A Periodontal bacterial invasion and infection: contribution to atherosclerotic pathology. J Periodontol (2013) 84(4 Suppl):S30–50 10.1111/jcpe.12079 23631583

[86] Chaparro A Blanlot C Ramirez V Sanz A Quintero A Inostroza C *Porphyromonas gingivalis*, *Treponema denticola* and toll-like receptor 2 are associated with hypertensive disorders in placental tissue: a case-control study. J Periodontal Res (2013) 48(6):802–9. 10.1111/jre.12074 23711357

[87] Chaparro A Sanz A Quintero A Inostroza C Ramirez V Carrion F Increased inflammatory biomarkers in early pregnancy is associated with the development of pre-eclampsia in patients with periodontitis: a case control study. J Periodontal Res (2013) 48(3):302–7. 10.1111/jre.12008 23035752

[88] Barak S Oettinger-Barak O Machtei EE Sprecher H Ohel G. Evidence of periopathogenic microorganisms in placentas of women with preeclampsia. J Periodontol (2007) 78(4):670–6. 10.1902/jop.2007.060362 17397314

[89] Swati P Thomas B Vahab SA Kapaettu S Kushtagi P. Simultaneous detection of periodontal pathogens in subgingival plaque and placenta of women with hypertension in pregnancy. Arch Gynecol Obstet (2012) 285(3):613–9. 10.1007/s00404-011-2012-9 21830010

[90] Moura da Silva G Coutinho SB Piscoya MD Ximenes RA Jamelli SR. Periodontitis as a risk factor for preeclampsia. J Periodontol (2012) 83(11):1388–96. 10.1902/jop.2012.110256 22309175

[91] Leon R Silva N Ovalle A Chaparro A Ahumada A Gajardo M Detection of *Porphyromonas gingivalis* in the amniotic fluid in pregnant women with a diagnosis of threatened premature labor. J Periodontol (2007) 78(7):1249–55. 10.1902/jop.2007.060368 17608580

[92] Dasanayake AP Boyd D Madianos PN Offenbacher S Hills E. The association between *Porphyromonas gingivalis*-specific maternal serum IgG and low birth weight. J Periodontol (2001) 72(11):1491–7. 10.1902/jop.2001.72.11.1491 11759860

[93] Sasahara J Kikuchi A Takakuwa K Sugita N Abiko Y Yoshie H Antibody responses to *Porphyromonas gingivalis* outer membrane protein in the first trimester. Aust N Z J Obstet Gynaecol (2009) 49(2):137–41. 10.1111/j.1479-828X.2009.00958.x 19441162

[94] Lin D Smith MA Elter J Champagne C Downey CL Beck J *Porphyromonas gingivalis* infection in pregnant mice is associated with placental dissemination, an increase in the placental Th1/Th2 cytokine ratio, and fetal growth restriction. Infect Immun (2003) 71(9):5163–8. 10.1128/IAI.71.9.5163-5168.2003 12933860 PMC187373

[95] Lin D Smith MA Champagne C Elter J Beck J Offenbacher S. *Porphyromonas gingivalis* infection during pregnancy increases maternal tumor necrosis factor alpha, suppresses maternal interleukin-10, and enhances fetal growth restriction and resorption in mice. Infect Immun (2003) 71(9):5156–62. 10.1128/IAI.71.9.5156-5162.2003 12933859 PMC187372

[96] Phillips P Brown MB Progulske-Fox A Reyes L *Porphyromonas gingivalis* strain specific effects on the placental bed. Am J Reprod Immunol (2014) 71(Suppl 1):75–6 10.1111/aji.12255

[97] Belanger M Reyes L von Deneen K Reinhard MK Progulske-Fox A Brown MB. Colonization of maternal and fetal tissues by *Porphyromonas gingivalis* is strain-dependent in a rodent animal model. Am J Obstet Gynecol (2008) 199(1):86.e1–7. 10.1016/j.ajog.2007.11.067 18355778

[98] Gomez-Lopez N Mial T Robertson SA Infection-induced preterm delivery does not involve a shift in macrophage polarization from the M2/M1phenotype but implicates a maternal pro-inflammatory state. Am J Reprod Immunol (2014) 71(Suppl 1):68 10.1111/aji.12255

[99] von Chamier M Allam A Brown MB Reinhard MK Reyes L. Host genetic background impacts disease outcome during intrauterine infection with *Ureaplasma parvum*. PLoS One (2012) 7(8):e44047. 10.1371/journal.pone.0044047 22952869 PMC3430619

[100] Allam AB von Chamier M Brown MB Reyes L Immune profiling of BALB/C and C57BL/6 mice reveals a correlation between *Ureaplasma parvum*-induced fetal inflammatory response syndrome-like pathology and increased placental expression of TLR2 and CD14. Am J Reprod Immunol (2014) 71(3):241–51 10.1111/aji.12192 24372928 PMC3927638

[101] Topping V Romero R Than NG Tarca AL Xu Z Kim SY Interleukin-33 in the human placenta. J Matern Fetal Neonatal Med (2013) 26(4):327–38 10.3109/14767058.2012.735724 23039129 PMC3563729

[102] Sharma S Kulk N Nold MF Graf R Kim SH Reinhardt D The IL-1 family member 7b translocates to the nucleus and down-regulates proinflammatory cytokines. J Immunol (2008) 180(8):5477–82. 10.4049/jimmunol.180.8.5477 18390730

[103] Li D Guabiraba R Besnard AG Komai-Koma M Jabir MS Zhang L IL-33 promotes ST2-dependent lung fibrosis by the induction of alternatively activated macrophages and innate lymphoid cells in mice. J Allergy Clin Immunol (2014). 10.1016/j.jaci.2014.05.011 24985397 PMC4258609

[104] Chaudhry SA Gad N Koren G. Toxoplasmosis and pregnancy. Can Fam Physician (2014) 60(4):334–6. 24733322 PMC4046541

[105] Tang Z Abrahams VM Mor G Guller S. Placental Hofbauer cells and complications of pregnancy. Ann N Y Acad Sci (2011) 1221:103–8. 10.1111/j.1749-6632.2010.05932.x 21401637 PMC3707113

[106] Toti P Arcuri F Tang Z Schatz F Zambrano E Mor G Focal increases of fetal macrophages in placentas from pregnancies with histological chorioamnionitis: potential role of fibroblast monocyte chemotactic protein-1. Am J Reprod Immunol (2011) 65(5):470–9. 10.1111/j.1600-0897.2010.00927.x 21087336 PMC3071455

[107] Vinnars MT Rindsjo E Ghazi S Sundberg A Papadogiannakis N. The number of CD68(+) (Hofbauer) cells is decreased in placentas with chorioamnionitis and with advancing gestational age. Pediatr Dev Pathol (2010) 13(4):300–4. 10.2350/09-03-0632-OA.1 19642814

[108] Ben Amara A Gorvel L Baulan K Derain-Court J Buffat C Verollet C Placental macrophages are impaired in chorioamnionitis, an infectious pathology of the placenta. J Immunol (2013) 191(11):5501–14. 10.4049/jimmunol.1300988 24163411

[109] Sisino G Bouckenooghe T Aurientis S Fontaine P Storme L Vambergue A. Diabetes during pregnancy influences Hofbauer cells, a subtype of placental macrophages, to acquire a pro-inflammatory phenotype. Biochim Biophys Acta (2013) 1832(12):1959–68. 10.1016/j.bbadis.2013.07.009 23872577

[110] Samaniego R Palacios BS Domiguez-Soto A Vidal C Salas A Matsuyama T Macrophage uptake and accumulation of folates are polarization-dependent in vitro and in vivo and are regulated by activin A. J Leukoc Biol (2014) 95(5):797–808. 10.1189/jlb.0613345 24399840

[111] Tang Z Buhimschi IA Buhimschi CS Tadesse S Norwitz E Niven-Fairchild T Decreased levels of folate receptor-beta and reduced numbers of fetal macrophages (Hofbauer cells) in placentas from pregnancies with severe pre-eclampsia. Am J Reprod Immunol (2013) 70(2):104–15. 10.1111/aji.12112 23480364 PMC3686834

[112] Ariza AC Bobadilla N Diaz L Avila E Larrea F Halhali A Placental gene expression of calcitonin gene-related peptide and nitric oxide synthases in preeclampsia: effects of magnesium sulfate. Magnes Res (2009) 22(1):44–9 10.1684/mrh.2009.0154 19441274

[113] Kim JS Romero R Kim MR Kim YM Friel L Espinoza J Involvement of Hofbauer cells and maternal T cells in villitis of unknown aetiology. Histopathology (2008) 52(4):457–64. 10.1111/j.1365-2559.2008.02964.x 18315598 PMC2896045

[114] Kim MJ Romero R Kim CJ Tarca AL Chhauy S LaJeunesse C Villitis of unknown etiology is associated with a distinct pattern of chemokine up-regulation in the feto-maternal and placental compartments: implications for conjoint maternal allograft rejection and maternal anti-fetal graft-versus-host disease. J Immunol (2009) 182(6):3919–27. 10.4049/jimmunol.0803834 19265171 PMC2754231

[115] Tamblyn JA Lissauer DM Powell R Cox P Kilby MD. The immunological basis of villitis of unknown etiology – review. Placenta (2013) 34(10):846–55. 10.1016/j.placenta.2013.07.002 23891153

